# A novel intermediate in transcription initiation by human mitochondrial RNA polymerase

**DOI:** 10.1093/nar/gkt1356

**Published:** 2014-01-06

**Authors:** Yaroslav I. Morozov, Karen Agaronyan, Alan C. M. Cheung, Michael Anikin, Patrick Cramer, Dmitry Temiakov

**Affiliations:** ^1^Department of Cell Biology, School of Osteopathic Medicine, Rowan University, Medical Center Dr, Stratford, NJ 08084, USA and ^2^Gene Center and Department of Biochemistry, Center for Integrated Protein Science Munich (CIPSM), Ludwig-Maximilians-Universität München, Feodor-Lynen-Strasse 25, 81377 Munich, Germany

## Abstract

The mitochondrial genome is transcribed by a single-subunit T7 phage-like RNA polymerase (mtRNAP), structurally unrelated to cellular RNAPs. In higher eukaryotes, mtRNAP requires two transcription factors for efficient initiation—TFAM, a major nucleoid protein, and TFB2M, a transient component of mtRNAP catalytic site. The mechanisms behind assembly of the mitochondrial transcription machinery and its regulation are poorly understood. We isolated and identified a previously unknown human mitochondrial transcription intermediate—a pre-initiation complex that includes mtRNAP, TFAM and promoter DNA. Using protein–protein cross-linking, we demonstrate that human TFAM binds to the N-terminal domain of mtRNAP, which results in bending of the promoter DNA around mtRNAP. The subsequent recruitment of TFB2M induces promoter melting and formation of an open initiation complex. Our data indicate that the pre-initiation complex is likely to be an important target for transcription regulation and provide basis for further structural, biochemical and biophysical studies of mitochondrial transcription.

## INTRODUCTION

Mitochondrial transcription does not fit the paradigm of eukaryotic or prokaryotic transcription systems, as it relies on a single subunit T7 phage-like mtRNAP. However, unlike the T7 system, transcription initiation by mtRNAP involves a number of additional factors, suggesting a more complex organization that likely reflects regulatory needs by the mitochondrial system (1,2).

Cellular multi-subunit polymerases form an array of transient complexes along the pathway to transcription initiation (3–7). These intermediates serve as important targets for regulation by presenting a specific conformation of RNAP to various regulatory factors. Binding of these factors at early stages of transcription is an important mechanism that affects cellular physiology and development, and this phenomenon has been well studied in a number of systems (8,9). As noted above, mtRNAP also requires auxiliary factors for transcription initiation; however, the mechanisms of promoter recognition, binding and melting by the mtRNAP must be distinct from those established for phage T7 RNAP (10–12), which does not require such factors, and in which formation of stable transcription intermediates has not been reported (13,14).

While most eukaryotic organisms contain mitochondria, the basal mitochondrial transcription machinery appears to have evolved differently in lower and higher eukaryotes. Thus, the yeast core transcription system is composed of mtRNAP and a single transcription initiation factor, Mtf1, which is implicated in promoter melting (15,16). In contrast, the mammalian core transcription apparatus contains, in addition to mtRNAP and TFB2M (a functional analog of Mtf1), an abundant mitochondrial protein, TFAM (17,18) that is a major component of the mitochondrial nucleoid and is required for mtDNA organization and maintenance; knockout of the latter protein results in a dramatic loss of mtDNA and disruption of oxidative phosphorylation (19,20). While yeast mitochondria also contain TFAM, it has no apparent role in transcription and serves only as a nucleoid protein, likely due to truncation (as compared with human TFAM) of a C-terminal ‘tail’ domain that has been implicated in transcription activation in human mitochondria (21). Human TFB2M is transiently associated with mtRNAP during initiation and interacts with the templating DNA base and the priming substrate (22). Both TFB2M and Mtf1 have been implicated in regulation of transcription initiation in response to variations in cellular ATP concentrations (22,23).

Despite recent progress in structural studies of human mtRNAP and TFAM–DNA complexes (2,24–26), the mechanisms of assembly of the mitochondrial transcription initiation complex are poorly understood and are somewhat controversial. It has been suggested that TFAM, which leaves a clear footprint on two major human mitochondrial promoters, termed LSP and HSP1 (21,27), interacts via its C-terminus with TFB2M, implicating the latter in recruiting mtRNAP to its promoter (28). On the other hand, it has been postulated that the mitochondrial core transcription system includes only mtRNAP and TFB2M, and that TFAM is dispensable for the initiation process and acts to stimulate basal levels of transcription from both the LSP and HSP1 promoters (29), and as a suppressor for a putative HSP2 promoter (30,31). However, both *in vivo* and *in vitro* transcription results, and the lack of situation where TFAM is absent from mitochondria *in vivo* argue that TFAM-independent initiation events are nonspecific and TFAM is a core component of the human mitochondrial transcription machinery (12,32,33).

In this work, we demonstrate that assembly of the mitochondrial transcription initiation complex occurs through formation of a distinct intermediate—a pre-initiation complex—that involves mtRNAP, TFAM and promoter DNA. We have isolated the pre-initiation complex, mapped interactions between its components and characterized functionally important regions in mtRNAP and promoter DNA. We also propose a molecular mechanism for TFAM action based on its direct interactions with mtRNAP and its recruitment to the promoter.

## MATERIALS AND METHODS

### Cloning, expression and purification of the components of human mitochondrial transcription

Cloning, expression and purification of TFAM and mtRNAP variants is described in Supplementary Methods.

### Transcription assays

Templates for transcription assays were prepared by polymerase chain reaction (PCR) or by annealing synthetic DNA oligo nucleotides as described in Supplementary Methods. Standard transcription reactions were carried out using synthetic or PCR DNA templates (50 nM), mtRNAP (50 nM), TFAM (50 nM), TFB2M (50 nM) in a transcription buffer containing 40 mM Tris (pH = 7.9), 10 mM MgCl_2_ and 10 mM dithiothreitol (DTT) in the presence of ATP (0.3 mM), GTP (0.3 mM), UTP (0.01 mM) and 0.3 µCi[α-^32^P] UTP (800 Ci/mmol) to produce 17–18 mer RNA products. Reactions were carried out at 35°C for the 30 min and stopped by addition of an equal volume of 95% formamide/0.05M EDTA. The products were resolved by 20% polyacrylamide gel electrophoresis (PAGE) containing 6 M urea and visualized by PhosphorImager (GE Health).

### Protein–protein cross-linking using 4-(N-maleimido)benzophenone

TFAM variants containing a single cysteine residue were treated with DTT (50 mM final) for 30 min at room temperature. The protein was then dialyzed against 40 mM HEPES (pH 7.0), 100 mM of NaCl for 2 h at 4°C. 4-(N-maleimido)benzophenone (MBP; Sigma, 1 mM solution in dimethylformamide (DMF)) was added to a dialyzed TFAM (500 µM) for 30 min at room temperature. The reaction was quenched by addition of DTT to a 5 mM final concentration and the modified TFAM was stored at −20°C. The initiation complexes (50 nM) were assembled using equimolar amount of DNA template, MBP-TFAM, ^32^P-labeled mtRNAP and/or ^32^P-labeled TFB2M and the cross-linking activated by ultraviolet (UV) irradiation (312 nm) for 5–10 min at room temperature.

### Protein–protein cross-linking using artificial photo reactive amino acid (pBpa)

The amber codon was introduced to TFAM or Δ119 mtRNAP gene using Quik Change site-directed mutagenesis kit (Agilent) as described above. Expression of pBpa-containing protein was performed as described previously (34) with modifications (see Supplementary Methods).

### Protein–DNA photo cross-linking

To generate template for protein–DNA photo cross-linking, a 5′ ^32^P-radiolabeled DNA primer with nonspecific sequence containing 4-thio UMP (Supplementary Figure S1) was annealed with nontemplate strand (69 nt) and template (49 nt) DNA strands containing LSP promoter sequence (−39 to +10). To increase efficiency of the cross-linking, a noncomplementary to 4-thio UMP nucleotide (CMP) was used in the nontemplate strand of the DNA (Supplementary Figure S1). Transcription ICs were formed as described above and UV irradiated (312 nm) for 15 min at room temperature in the presence of nonspecific oligonucleotides (10 µM). Cross-linking products were resolved using a 4–12% Bis–Tris NuPAGE gel (Invitrogen) and visualized by PhosphorImager^TM^ (GE Health).

### Mapping of the cross-linking sites in mtRNAP

Mapping of the TFAM interacting regions in mtRNAP with CNBr and NTCB (2-nitro-5-thiocyano-benzoic acid) was performed as described previously (35). Hydroxylamine (NH_2_OH) cleavage was performed as in (22), with modifications (see Supplementary Methods). In LysC mapping experiments, 2–4 ng of LysC protease (Sigma) was added to the cross-link reaction (10 µl) for 15–60 min at room temperature.

## RESULTS

### TFAM is required for transcription initiation and makes direct contacts with mtRNAP

**Figure 1.**
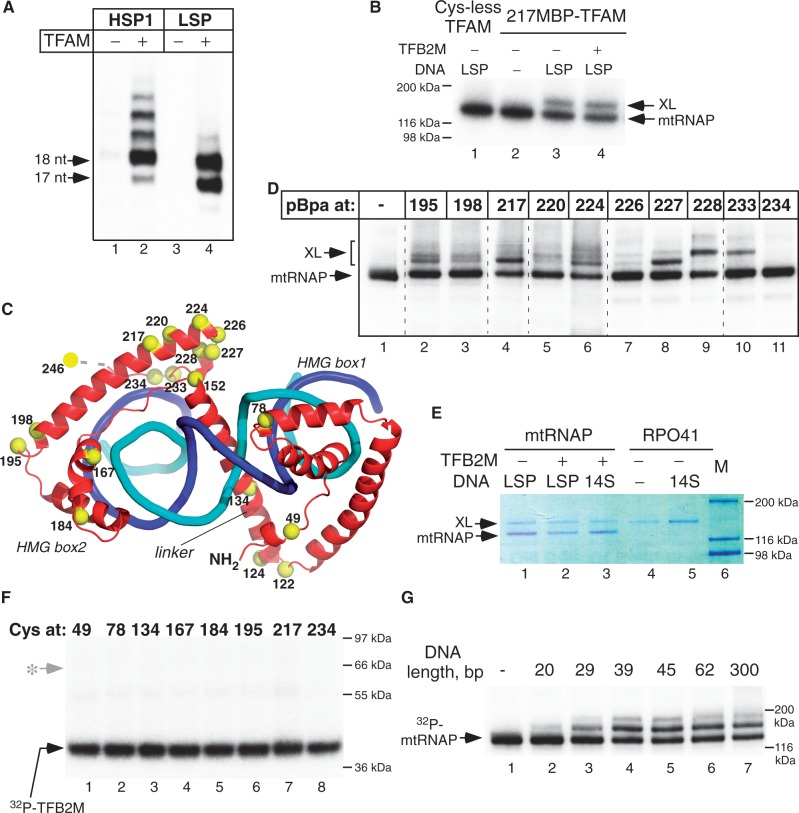
TFAM makes direct interactions with mtRNAP. (**A**) TFAM is absolutely required for efficient transcription of both mtDNA promoters. *In vitro* transcription assay was performed with the nucleotide sets lacking CTP using PCR amplified templates with the HSP1 (lanes 1,2) or LSP (lanes 3,4) promoters. The gel image is overexposed to dramatize lack of transcription initiation on the LSP and trace activity (<0.5%) observed on the HSP1 in TFAM absence. (**B**) TFAM-mtRNAP interactions do not require TFB2M but depend on the DNA presence. The complexes were assembled using MBP-modified Cys217TFAM and ^32^P-labeled mtRNAP and TFB2M (where indicated) and UV-irradiated in the absence (lanes 1,2) or in the presence (lanes 3,4) of DNA. (**C**) Location of the residues probed in photo cross-linking experiments using MBP or pBpa (yellow spheres) on TFAM-DNA structure. (**D**) Scanning cross-linking of pBpa-containing TFAM and mtRNAP. The pre-initiation complexes were assembled using ^32^P-labeled mtRNAP (50 nM), 50 nM LSP and 50 nM TFAM having pBpa at the position indicated, UV irradiated and resolved in SDS-PAGE. Note that covalently linked polypeptides may migrate differently depending on the point of attachment. (**E**) TFAM does not cross-link to the heterologous mtRNAP. Pre-initiation complexes were assembled using MBP-modified Cys217TFAM and mtRNAP (lanes 1–3) or yeast mtRNAP (RPO41) (lanes 4,5) in the absence or presence of DNA (LSP for human mtRNAP and 14S promoter for RPO41), as indicated and UV-irradiated. Molecular weight markers are shown in lane 6. Note that molecular weight of RPO41 (155 kDa) is similar to that of TFAM-mtRNAP cross-link. (**F**) TFAM does not cross-link to TFB2M. Initiation complexes (150 nM) were assembled using mtRNAP, ^32^P-labeled TFB2M, MBP-modified TFAM and the LSP promoter. The grey arrow with an asterisk marks the expected position of the TFB2M-TFAM cross-linking species. (**G**) TFAM-mtRNAP interactions require DNA long enough to accommodate both proteins. Cross-linking was performed using Cys217MBP-TFAM and WT RNAP and synthetic double-stranded DNA having nonspecific sequence and the lengths indicated.

Previous studies suggesting a stimulatory effect of TFAM on mitochondrial transcription *in vitro* used both the LSP and HSP1 promoters (12,21,27,29); however, at certain conditions nonspecific transcription events have been observed when TFAM was absent from reaction (32). In our study, we minimized the effects of sequence context among different templates using PCR-amplified promoter fragments of similar lengths having identical sequences downstream of the transcription start sites (Figure 1A and Supplementary Figure S1). The results demonstrate that there is a dramatic increase in transcription from both promoters in the presence of TFAM, confirming its critical role in transcription stimulation.

To examine further the mechanism of transcription stimulation by TFAM, we probed molecular interactions within a transcription initiation complex (IC) composed of promoter DNA, mtRNAP, TFAM and TFB2M by protein–protein cross-linking methods. Our approach used TFAM variants having single photo-reactive probe at various positions, and a ^32^P-labeled ‘bait’ protein [modified mtRNAP or TFB2M that included an engineered protein kinase (PKA) phosphorylation site] (Supplementary Figure S2). On assembly of the IC and activation of the cross-link by UV irradiation the covalently bound species were resolved by PAGE and detected by autoradiography. Two cross-linking strategies were used (Supplementary Figure S3). In the first, a bi-functional cross-linker (N-maleimido)-benzophenone (MBP) was used to modify single cysteine residues within TFAM that had been introduced at specific positions (Supplementary Figure S3). In the second, a photo reactive amino acid residue, para-benzoyl phenylalanine (pBpa), was incorporated at specific positions in TFAM during expression in *Escherichia coli* (34) (Supplementary Figure S3). Efficient pBpa cross-linking occurs within 3–4 Å from the nearest carbon atom of the target protein (36), as opposed to an MBP derivative, which can interact at distances of up to 10 Å and was advantageous during initial rounds of probing protein–protein interactions (37).

During initial experiments we identified a number of MBP and pBpa-modified TFAM variants that cross-linked to mtRNAP, as evidenced by the appearance of a high molecular weight species that corresponded to the size of mtRNAP plus TFAM (∼160 kDa; Figure 1B,C and Supplementary Figure S4). The most efficient cross-linking was observed when the probes were located in the C-terminal region of TFAM with the 217MBP- or 217pBpa-TFAM, producing 50–60% cross-linking (Figure 1B–D). Notably, the cross-link between mtRNAP and TFAM required the presence of DNA but was TFB2M-independent (Figure 1B, lanes 1–4), indicating formation of a transcription intermediate—a pre-initiation complex (pre-IC). Subsequent scanning of TFAM with probes introduced at other locations revealed several points in the C-terminal region (residues 227, 228 and 233) that also resulted in highly efficient cross-linking, suggesting a proximity of this region of TFAM to mtRNAP (Figure 1D).

To determine whether the TFAM-mtRNAP cross-link was specific, we used yeast *Saccharomyces cerevisiae* mtRNAP in the cross-linking experiments (Figure 1E). No cross-linking between heterologous RNAP and TFAM was observed, indicating that interactions between TFAM and mtRNAP in the pre-IC may be species-specific. Previous reports had indicated that TFAM interacts with TFB2M (28). We therefore repeated the cross-linking experiments using ^32^P-labeled TFB2M and all available MBP and pBpa-modified TFAM variants (Figure 1C and F and Supplementary Tables S1, S2). No TFAM-TFB2M cross-link was detected with any of these variants.

We next determined the optimal length of DNA required for efficient TFAM-DNA cross-linking using DNA templates having nonspecific sequences and found that at least 39–45 bp of DNA were necessary to provide efficient cross-linking (Figure 1G). Considering the size of the TFAM (20 bp) and mtRNAP (20–25 bp) footprints on DNA (22,27), this suggests that the interaction of TFAM and mtRNAP also requires their association (albeit nonspecific) with DNA. We observed no significant difference in mtRNAP-TFAM cross-linking efficiency on LSP, HSP1 or nonspecific DNA (Supplementary Figure S5) in heparin and salt-challenge experiments. These results reflect the cumulative property of the photo cross-linking technique and the transient nature of the complex and will be discussed further below.

### TFAM interacts with the N-terminal domain of mtRNAP in the pre-IC

To map the site(s) in mtRNAP that interact with TFAM, we cross-linked N-terminal ^32^P-labeled mtRNAP to TFAM in the presence of DNA (as described above) and used a series of specific proteases to generate a nested set of N-terminal ^32^P-labeled mtRNAP peptides using NTCB (2-nitro-5-thiocyano-benzoic acid, cleaves at cysteine residues), hydroxylamine (cleaves between asparagine and glycine residues) and Lys C protease (cleaves at lysines) (35,38). Peptides that are cross-linked to TFAM are expected to have an increased molecular weight and appear shifted (compared with the uncross-linked peptides) on sodium dodecyl sulphate (SDS)-PAGE analysis. To simplify interpretation of the cleavage pattern in these experiments, we used an N-terminal deletion mutant of mtRNAP (Δ119, residues 120–1230) that possesses all of the properties of WT mtRNAP in transcription initiation assays.

**Figure 2.**
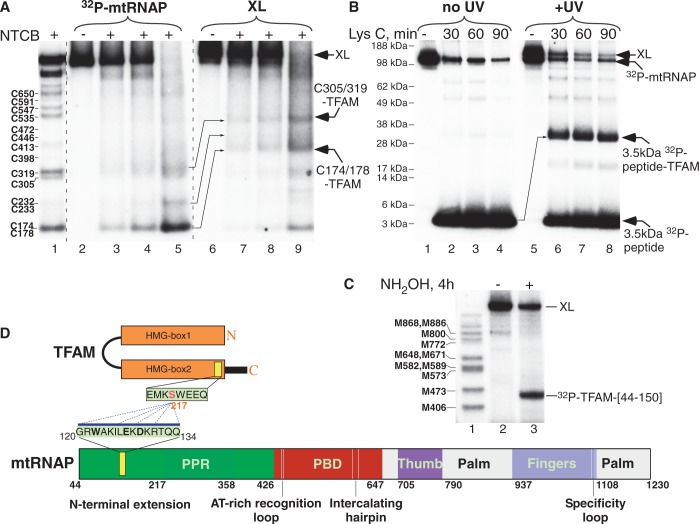
TFAM interacts with the N-terminal region of mtRNAP. (**A**) Mapping of TFAM-mtRNAP cross-link with NTCB. ^32^P-labeled Δ119 mtRNAP was treated with NTCB to generate a set of peptide markers (lane 1). The pre-IC (50 nM) was assembled with ^32^P-labeled Δ119 mtRNAP and 217MBP-TFAM and UV irradiated. The cross-linked species (lanes 6–9) were separated from the free mtRNAP (lanes 2–5) and treated with NTCB for 5 (lanes 3,7), 10 (lanes 4,8) or 15 (lanes 5,9) min. The residual low molecular bands in lanes 6–9 likely represent de-cross-linking taking place during the electro-elution procedure. (**B**) Fine mapping of TFAM-mtRNAP cross-link with LysC. The pre-IC was assembled as described above and treated with LysC protease for the time indicated before (lanes 2–4) and after (lanes 6–8) UV-irradiation. The 3.5 kDa peptide visible on Lys C cleavage corresponds to the very N-terminus of mtRNAP (sequence MGHHHHHHRRASVGRWAKILEKDKRTQQMRMQRLK, the PKA site is underlined). (**C**) Mapping of 217pBpa-TFAM cross-linking region in mtRNAP with hydroxylamine. The cross-linked pre-IC (lane 2) was treated with hydroxylamine for 4 h (lane 3) and the products of the reaction resolved using SDS-PAGE. Radioactive protein markers (lane 1) were generated using CNBr cleavage of ^32^P-labeled mtRNAP. (**D**) Schematics of the cross-link mapping data illustrating regions of mtRNAP–TFAM interactions.

To map the region in mtRNAP that interacts with TFAM cross-linked at position 217 (which gave the most efficient cross-link), we used cleavage with NTCB (Figure 2A and Supplementary Figure S6A). Cross-linked species were separated from uncross-linked mtRNAP by SDS-PAGE and, after electro-elution, were treated with NTCB (Figure 2A). The lowest band on the NTCB cleavage pattern observed with uncross-linked Δ119 mtRNAP corresponds to cleavage of the peptide bond at the two most N-terminal mtRNAP cysteine residues, Cys 174 and Cys 178 (Figure 2A, lanes 2–5). When TFAM-mtRNAP cross-linked at position 217 was treated with NTCB, the labeled peptides, including the smallest one generated by cleavage at Cys 174/178, were shifted up by one TFAM mass (Figure 2A, lanes 6–9). This indicates that the TFAM cross-linking region in mtRNAP is located in the N-terminus of mtRNAP, between residues 120 and 174/178.

To narrow the cross-linking region, we used proteolysis by LysC protease. LysC treatment of the uncross-linked ^32^P-labeled Δ119 mtRNAP rapidly generated a 3.5 kDa peptide (Figure 2B, lanes 1–4). This peptide was also radiolabeled and hence contained the engineered PKA site (Supplementary Figure S2) indicating that it corresponds to the N-terminal region of mtRNAP. Cleavage of cross-linked mtRNAP-TFAM resulted in new species that corresponds to TFAM covalently attached to the N-terminal 3.5 kDa fragment of mtRNAP (Figure 2B, lanes 5–8). Based on the size of the N-terminal peptide, we conclude that the site of 217TFAM cross-linking is located between residues 120–141/143 in the N-terminal extension domain of mtRNAP, in agreement with the NTCB mapping data above.

Finally, to verify the NTCB and LysC mapping data we used hydroxylamine cleavage (Figure 2C and Supplementary Figure S6B). Treatment of the cross-link obtained with ^32^P-labeled TFAM and mutant mtRNAP variant containing a single hydroxylamine cleavage site at position 150 (NG150) results in appearance of a labeled fragment (37 kDa) that represents the region 44–150 of mtRNAP, consistent with the mapping data above. To summarize, the results of our mapping studies suggest that TFAM interacts with the N-terminal extension region of mtRNAP (residues 120–143) (Figure 2D). This region of mtRNAP is apparently flexible and was not resolved in the crystal structures of mtRNAP (2,39).

### TFAM interacts with functionally important region of mtRNAP

**Figure 3.**
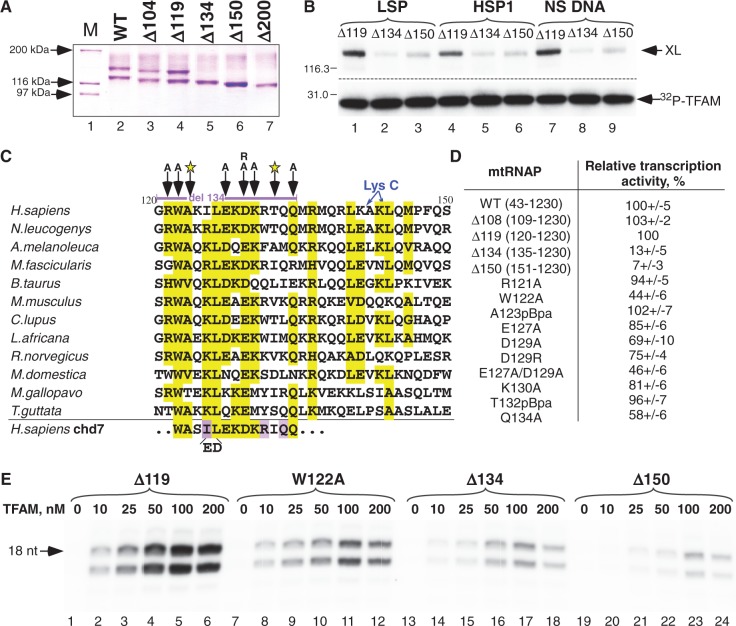
TFAM interaction region in mtRNAP. (**A**). Cross-linking of TFAM with mtRNAP deletion mutants. The cross-linking was performed using Cys217MBP-TFAM and the mtRNAP mutants indicated in the presence of the LSP promoter. (**B**) Importance of 120–134 region of mtRNAP for TFAM interactions. Cross-linking was performed using ^32^P-labeled Cys217MBP and the mtRNAP mutants indicated on LSP, HSP1 or nonspecific (NS) DNA template. (**C**) Sequence conservation in the TFAM-binding region of mtRNAP of different mammalian and avian species. Black arrows and letters indicate point mutations made in this region of mtRNAP. The star indicates substitutions to pBpa. Lysine residues cleaved by LysC are marked by blue arrows. (**D** and **E**) Relative transcription activity of mtRNAP mutants having deletions or substitutions in the region of TFAM binding. *In vitro* transcription initiation assay was performed as described in ‘Materials and Methods’ section using the LSP template.

To analyze the functional importance of the TFAM-binding regions we constructed a series of N-terminal mtRNAP deletion mutants. We found that although efficient TFAM cross-linking was observed with WT, Δ104 and Δ119 mtRNAP, no cross-linking was detected when larger fragments (Δ150 and Δ200) were removed from the N-terminal domain (Figure 3A and B). An additional deletion mutant, Δ134 mtRNAP, was constructed to narrow down this functionally important region (Figure 3B) and the TFAM binding region was localized to residues 120–134, consistent with the mapping data above. Sequence analysis of mammalian mtRNAPs reveals high homology in this region (Figure 3C). A number of charged and hydrophobic residues are conserved, with W122 and L126 residues being invariant in birds and mammals. We also noted that in the TFAM-binding region of mtRNAP, 11 residues are identical to the region found in the human chromodomain helicase DNA binding protein 7 (chd7); however, the significance of this sequence similarity is unclear.

To further probe the functional importance of the TFAM-binding region in mtRNAP we assayed the transcription properties of the N-terminal deletion mtRNAP mutants as well as mutants in which conserved residues in this interval were substituted (Figure 3C and D). MtRNAP variants Δ134 and Δ150 were unable to support transcription on native DNA (Figure 3D), but exhibited activity on pre-melted promoter templates transcription of which is TFAM and TFB2M-independent (22) (Supplementary Figure S7A). Most single-residue substitution mtRNAP mutants showed a modest (1.5–2-fold) reduction of transcription initiation (Figure 3D).

**Figure 4.**
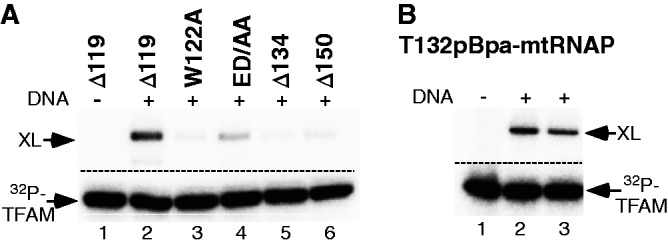
Functional activity of mtRNAP mutants. (**A**). Substitutions within mtRNAP TFAM-binding region result in a loss of cross-linking efficiency. The pre-ICs (50 nM) were assembled using ^32^P-labeled 217MBP-TFAM and Δ119 (lanes 1,2) or mutant mtRNAP (lanes 3–6) as indicated and UV-irradiated. The cross-linking species were separated using 10% SDS-PAGE. (**B**). MtRNAP having pBpa in the TFAM-binding region cross-links to TFAM. The pre-ICs were assembled using ^32^P-labeled TFAM (50 nM) and T132pBpa-mtRNAP (50 nM, lane 2, and 100 nM, lane 3), UV-irradiated for 5 min and analyzed as described above.

Notably, substitution of the invariant W122 residue (W122A) and a double mutation involving conserved negatively charged residues E127 and D129 (E127A/D129A) exhibited a 2–2.5-fold reduction of transcription activity (Figure 3E and Supplementary Figure S7B). These data suggest that binding of TFAM to the N-terminal extension region of mtRNAP likely involves a combination of hydrophobic and electrostatic interactions. Interestingly, when we assayed the cross-linking efficiency of the W122A and ED/AA mutants we found that the former was unable to produce an efficient cross-link when probed with Cys217MBP-TFAM, suggesting that the W122 residue is a primary target for the cross-linking (Figure 4A).

Finally, to confirm the cross-linking data and to identify key residues in TFAM involved in interactions with mtRNAP, we introduced a pBpa residue in the TFAM-binding region of mtRNAP at position 132 (Figure 4B). Efficient DNA-dependent cross-linking with ^32^P-labeled TFAM was observed, confirming that this region of mtRNAP interacts with TFAM.

### MtRNAP interacts with the far upstream promoter region

**Figure 5.**
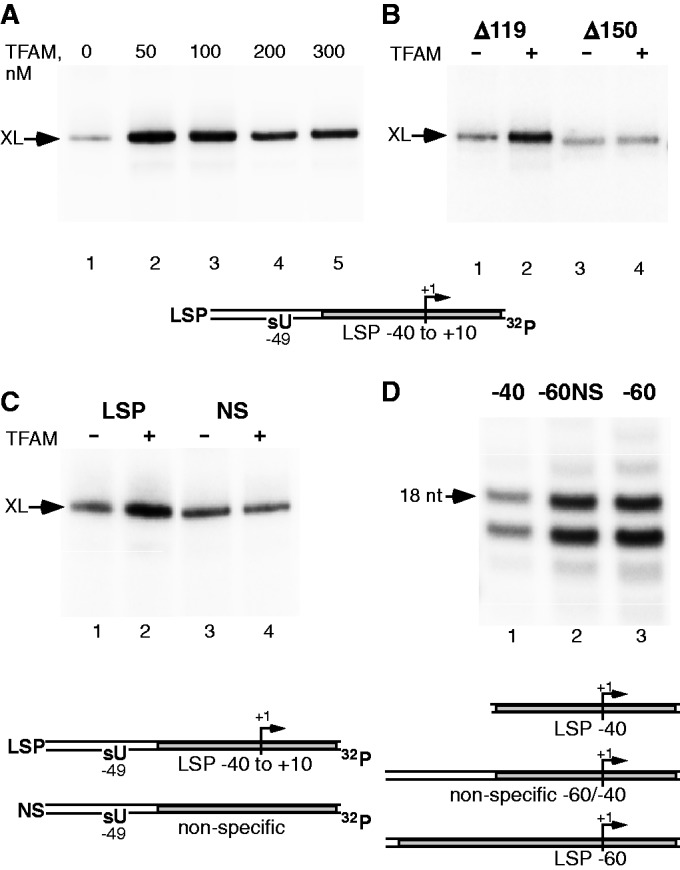
The upstream promoter DNA region contours mtRNAP molecule in the pre-IC. (**A**) DNA cross-linking at −49 base is TFAM-dependent. Pre-initiation complexes (150 nM of ^32^P-labeled template containing 4-thio UMP at position −49, 150 nM mtRNAP and 0–400 nM TFAM) were UV-irradiated for 15 min. (**B**) Mutant mtRNAP lacking N-terminal TFAM-binding site does not cross-link to the upstream promoter DNA. The cross-link was performed using WT or Δ150 mtRNAP in the presence (lanes 2,4) or absence (lanes 1,3) of TFAM by UV-irradiation for 15 min. (**C**) Promoter sequence is required for DNA-TFAM cross-linking. The reaction contained WT mtRNAP, TFAM (where indicated) and templates with (lanes 1,2) or without (lanes 3,4) LSP promoter sequence. (**D**) The far upstream promoter region (−60 to −40) is important for efficient transcription. Transcription activity was measured using synthetic template having LSP promoter from −40 (‘−40’, lane 1), nonspecific sequence from −40 to −60 (‘−60NS’, lane 2) or LSP promoter from −60 (‘−60’, lane 3).

Our cross-linking and functional data indicate that TFAM binds mtRNAP only in the presence of DNA and that this process is TFB2M-independent. We therefore propose that a transient transcription intermediate (a pre-IC) must exist along the transcription initiation pathway. Taking into account recent structural data demonstrating an extreme bending of promoter DNA around TFAM (17,25), we hypothesized that mtRNAP may be sandwiched between two DNA duplexes representing the downstream and upstream promoter regions. To test this we probed the association of mtRNAP with DNA using the photo reactive cross-linking nucleotide 4-thioUMP incorporated at the −49 position of the template strand of a radiolabeled LSP promoter (Figure 5A). The DNA-mtRNAP cross-link was efficient only in the presence of TFAM and its maximal efficiency was observed at a 1:1 ratio of polymerase and TFAM, as expected from the stoichiometry of the pre-IC. To further confirm that upstream DNA–mtRNAP interactions depend on TFAM, we used a mutant Δ150 mtRNAP that lacks the TFAM binding region and cannot form a pre-IC (Figure 5B). In the absence of TFAM both Δ119 and Δ150 mtRNAPs produced nonspecific cross-links (lanes 1 and 3). Addition of TFAM notably increased cross-linking of DNA to Δ119 mtRNAP but not to Δ150 mtRNAP, confirming that formation of the pre-IC is required for interaction of mtRNAP with the upstream promoter region. In addition, we analyzed mtRNAP–DNA interactions using a DNA template lacking a promoter sequence and found no specific (i.e. TFAM-dependent) cross-linking (Figure 5C).

The proximity of the far upstream DNA region to polymerase suggests that there could be important but previously overlooked interactions with mtRNAP in this region. When we compared synthetic promoter templates having 40 or 60 bp upstream of the promoter start site, we found that the latter exhibited a 2–3-fold increased activity (Figure 5D, lanes 1–2). To address the specificity of these interactions, an LSP template containing nonspecific DNA sequence in the region −60 to −40 was used. However, a template with the −60 LSP region (Figure 5D, lane 3) demonstrated no improvement over the template with a random −60/−40 region (Figure 5D, lane 2). These data suggest that it is proximity to DNA rather than the sequence itself that plays a role in stabilization of the initiation events and that the functional definition of the HSP1 and LSP promoters should be extended beyond the TFAM footprint to include the −60 to −40 region. These findings are also in an excellent agreement with the TFAM-mtRNAP foot-printing data (accompanying manuscript, Posse *et al.*, *Nucleic Acids Res* 2013).

## DISCUSSION

**Figure 6.**
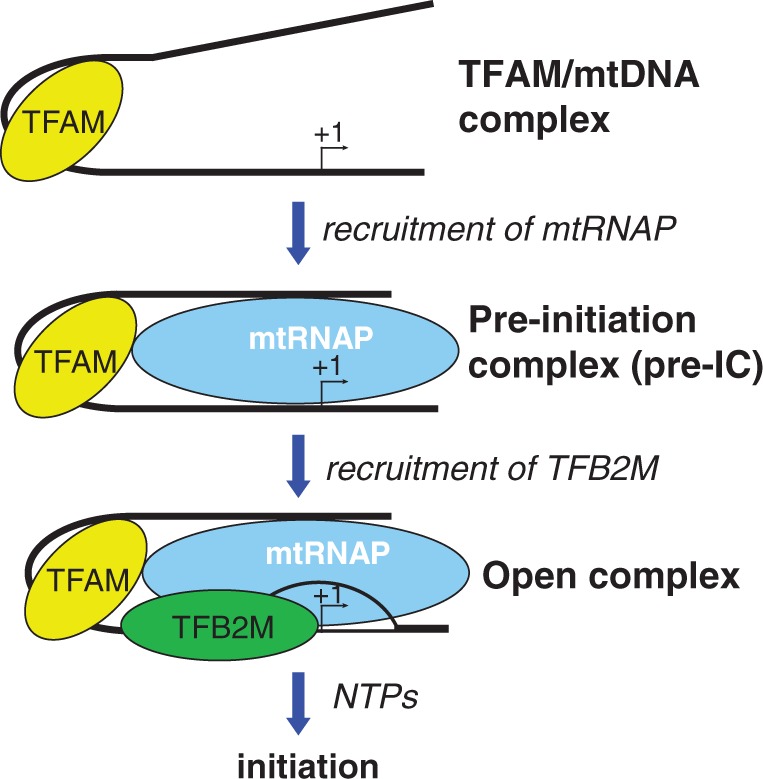
Assembly of transcription initiation complexes in human mitochondria. Binding and bending of DNA allows for TFAM and mtRNAP interaction and recruitment of the latter to the promoter where it forms a pre-initiation complex. The pre-IC, in turn, recruits TFB2M, which is required for promoter melting and initiation of RNA synthesis.

Interactions between the AT-rich recognition loop and the upstream promoter region are important features of transcription initiation by phage RNAPs (40,41). It is likely that lack of these interactions renders mtRNAP less specific and decreases its affinity to the promoter (2). Tight binding of TFAM to its recognition sequence at a defined position relative to the promoter start site, coupled with its association with mtRNAP, compensate for these changes, and appear to be crucial conditions for *de novo* RNA synthesis in mammalian mitochondria (Figure 6). Thus, recruitment of mtRNAP by TFAM results in formation of a transcription intermediate (the pre-IC), in which TFAM contributes to promoter selectivity and binds mtRNAP by establishing an interacting interface and bending the upstream promoter DNA around mtRNAP. Subsequent initiation events require TFB2M for promoter melting and formation of an open IC and NTP binding (22) (Figure 6). Importantly, we detected no TFAM-TFB2M interaction during transcription initiation in our cross-linking experiments. We also did not detect TFAM–TFB2M interactions in band-shift assays (not shown). Taken together these data suggest that in the IC these proteins do not contact each other. This is in contrast to a previous study that reported direct TFAM–TFB2M interaction based on a solid phase protein–protein binding assay (28). In this assay, TFB2M (or TFB1M) that has been immobilized on beads using an affinity tag, retained the full-length TFAM but not a TFAM variant lacking the C-terminal 10 amino acids. This retention had been presented as an evidence of interaction between TFAM and TFB2M (or TFB1M). However, an essential control had not been demonstrated—binding of TFAM to the column in the absence of TFB2M. Moreover, the finding that TFAM mutant lacking the C-terminal 10 amino acids is fully active in transcription (21) suggests that this putative TFB2M-TFAM interaction is functionally irrelevant.

The suggested mechanism of transcription initiation in human mitochondria (Figure 6) also implies that initiation events may involve formation of many pre-ICs along the mitochondrial DNA owing to the nonspecific (i.e. nonpromoter) binding capability of TFAM. In this scenario, the transient nature of TFAM-polymerase interactions and/or sliding of TFAM along the mtDNA recently suggested by Wuite and colleagues (42) may contribute to promoter selection in a way reminiscent of phage or bacterial RNAP—by enhancing lateral diffusion of mtRNAP along the DNA until the promoter is found. Alternatively, mtRNAP can be recruited by TFAM already bound to the promoter region. Since TFAM bends the promoter to a greater degree than a nonspecific DNA and this bending is C-terminal tail-dependent (43), only the pre-ICs that are formed on LSP or HSP1 may become competent to recruit TFB2M, resulting in specificity of transcription initiation.

The major finding of this study—identification of a novel transcription intermediate—suggests that, though mtRNAP belongs to a class of single-subunit polymerases, the basic principles of assembly of mitochondrial transcription initiation complexes are surprisingly similar to other cellular transcription systems. Moreover, by analogy with the transcription initiation process in prokaryotes and eukaryotes where promoter binding and melting are highly regulated events (3,9,44,45), the pre-IC may also serve as an important point of regulation of transcription in mitochondria. Thus, the rates of transcription initiation that depend on stability of the assembled pre-ICs can be affected by phosphorylation of TFAM, which has been demonstrated to decrease its binding to promoter DNA (46). It is also possible that another transcription factor, TFB2M, a transient component of mtRNAP catalytic site, would ‘sense’ a particular conformation of the pre-IC, either in the presence of another factor or when ATP concentration is changed (22,23). Likewise, the closed complexes assembled on different bacterial promoters are subject to regulation by transcription factors, such as DksA, which functions in conjunction with NTPs and/or ppGpp (47).

Interactions of TFAM with mtRNAP during transcription initiation also suggest that the strategy behind promoter escape mechanisms in mtRNAP is likely similar to the one observed in structurally unrelated multi-subunit cellular RNAPs. Recent structural data suggest that unlike situation with a related phage T7 RNAP, mtRNAP promoter binding domain does not undergo refolding during transition to the elongation stage of transcription (39) and therefore mtRNAP likely relies on release of TFAM and TFB2M for promoter clearance.

Finally, the extreme DNA bending by TFAM on both LSP and HSP1 (which are just 150 bp apart in human DNA) and the interactions of mtRNAP with the upstream promoter regions impose remarkable restriction on topology of the transcription initiation unit. Similarly, binding of nucleoid proteins such as Fis and IHF to bacterial promoter alters its topology and regulates bacterial transcription by facilitating promoter–RNAP interactions (48,49). As a result of such topological changes in mtDNA, assembly of two transcription pre-initiation complexes occurs in close proximity to each other. It is tempting to speculate that such proximity may provide an opportunity for regulation of transcription initiation events (and perhaps replication) on both promoters by yet-unidentified mitochondrial transcription factor(s).

## SUPPLEMENTARY DATA

Supplementary Data are available at NAR Online.

## FUNDING

National Institutes of Health (NIH) [RO1GM104231 to D.T.]; the Deutsche Forschungsgemeinschaft, [SFB646, SFB960, TR5, GRK1721, CIPSM, NIM, QBM]; the BioImaging Network; an ERC Advanced Grant; the Jung-Stiftung and the Vallee Foundation (to P.C.). Funding for open access charge: NIH.

*Conflict of interest statement*. None declared.

## Supplementary Material

Supplementary Data
