# Supplementary material for: A novel intermediate in transcription initiation by human mitochondrial RNA polymerase
Source: Nucleic Acids Res. 2014 Jan 6;42(6):3884–93. doi: 10.1093/nar/gkt1356 (PMC3973326; doi:10.1093/nar/gkt1356)
Supplement: Supplementary Data [file supp_42_6_3884__index.html]

A novel intermediate in transcription initiation by human mitochondrial RNA polymerase — A novel intermediate in transcription initiation by human mitochondrial RNA polymerase — Supplementary Data 

# A novel intermediate in transcription initiation by human mitochondrial RNA polymerase

## Supplementary Data

files

**Files in this Data Supplement:**

- Supplementary Data - pdf file
