# Supplementary material for: A novel intermediate in transcription initiation by human mitochondrial RNA polymerase
Source: Nucleic Acids Res. 2014 Jan 6;42(6):3884–93. doi: 10.1093/nar/gkt1356 (PMC3973326; doi:10.1093/nar/gkt1356)
Supplement: Supplementary Data [file supp_gkt1356_nar-03297-h-2013-File008.pdf]

Supplemental Material

A Novel Intermediate in Transcription Initiation by Human Mitochondrial RNA Polymerase

Yaroslav I. Morozov, Karen Agaronyan, Alan C.M. Cheung, Michael Anikin, Patrick Cramer and Dmitry Temiakov

Includes Extended Materials and Methods, figures S1-S7 and tables S1,S2.

Extended Materials and Methods

Cloning, expression and purification of the components of human mitochondrial transcription

The coding sequences of human TFAM (without leader peptide, residues 43-246), was amplified by PCR from human heart cells cDNA (Panomics) and cloned into the expression vector pET22b (Invitrogen), that allowed expression of N-terminally His6-tagged version of a protein. The coding sequence of human mtRNAP variant lacking first 119 residues (Δ119 mtRNAP) was amplified by PCR and cloned into the expression vector pProEx(Hb) (Invitrogen). Expression and purification of WT (wild type) mtRNAP and TFB2M was described previously (*Sologub et al, Cell 2009*). TFAM was expressed using BLR cells (DE3, recA, Novagen) to prevent homologous recombination with *E.coli* plasmid resulting in a truncated and thus inactive form of TFAM. TFAM was purified using affinity purification on Ni agarose and heparin-sepharose and then using anion exchange chromatography on Mono S column.

Construction of TFAM and mtRNAP mutants

TFAM mutants were obtained by site-directed mutagenesis (Quick-change, Agilent) starting with the plasmid containing N-terminal His-tagged WT TFAM (see above). TFAM variants containing phosphorylation site for PKA kinase (NEB) were made by insertion of residues RRA after serine 43. Cysteine-less TFAM variant was obtained by introduction of C46S substitution and deletion of C246 residue in a WT background. Mature form of mtRNAP with the PKA site was obtained by insertion of residues ASV after R54 by site-directed mutagenesis (Figure S2). PKA site in Δ119 mtRNAP was introduced by insertion of residues RRSVA after His6 tag. All subsequent mutants were made using Δ119 mtRNAP or Δ119 PKA mtRNAP (Figure S2). MtRNAP mutant used in photo cross-linking and mapping experiments (NG150 mtNRAP) was constructed using NG-less WT mtRNAP (residues 44-

1230) described previously (22). Activity of NG150 mtRNAP mutant in primer extension assays was found to be similar to the activity of the WT mtRNAP.

Promoter templates for transcription assays

Templates for transcription assays were prepared by PCR amplification of pT7blue plasmid containing -70 to +70 of native LSP sequence. PCR template with the HSP1 promoter contained native HSP1 sequence from -70 to +5 and a downstream sequence (from +6 to +70) identical to that of LSP promoter. Thus, both LSP and HSP1 templates contained identical initially transcribed sequence. The products of the PCR amplification (369 bps total, 144 bps of the transcribed sequence) were purified using the PCR clean-up kit (Qiagen) and eluted with water. Sequence of the synthetic templates with LSP or HSP1 promoters is shown in Figure S2.

Protein-protein cross-linking using artificial photo reactive amino acid (pBpa).

BLR expression cells were co-transformed with mtRNAP or TFAM expression plasmid (Ampr) and and pEVOL (Cmr) plasmid for pBpa incorporation. Cells were grown in LB media, supplemented with 50 μg/ml ampicillin and 30 μg/ml chloramphenicol at 37^0C until OD_{600} reached 0.8. The protein expression was induced by addition of IPTG (0.5 mM), arabinose (0.02%) and pBpa (Bachem, 1 mM) at 16^0C for 16h. The purified pBpa-containing proteins were used to assembled pre-ICs or ICs as described above and cross-linking activated by UV irradiation at 312 mn for 15 min at room temperature.

Mapping of the cross-linking sites in mtRNAP

For hydroxylamine (NH_2OH) cleavage, 2-10 μl of the cross-linked material was lyophilized, dissolved in 30 μl of solution containing 2M NH_2OH, 0.2M Na_2CO_3 and 4M Guanidine (pH 10-11.5 adjusted with 50% NaOH), and incubated for 2-4 h at 43^0C. The reaction was stopped by addition of 70 μl of H_2O and 400 μl of acetone, the products precipitated at -70^0C for 30 min, re-suspended in SDS gel loading buffer and resolved in 4-12% Bis-Tris NuPAGE gel (Invitrogen).

LSP promoter (non-template strands, 5' to 3')

+1

```
                    GTGTTAGTTGGGGGGTGACTGTTAAAAGTGCATACCGCCAAAAGATAAAATTTGTGGGCC   hLSP-40
GAGTGGGAGGGGAAAATAATGTGTTAGTTGGGGGGTGACTGTTAAAAGTGCATACCGCCAAAAGATAAAATTTGTGGGCC   hLSP-60
GCGGCGATCTCGATTCAGACGTGTTAGTTGGGGGGTGACTGTTAAAAGTGCATACCGCCAAAAGATAAAATTTGTGGGCC   LSP-40-60NS
```

HSP1 promoter (non-template strand, 5’ to 3’)

+1

```
CCATCCTACCCAGCACACACACACCGCTGCTAACCCCATACCCCGAACCAACCAAACCCCAAAGACACCCGCCACAGTTTAAA   h60NT1 WT
```

Templates for DNA cross-linking experiments

LSP promoter template

+1

```
 5'GCGGCGATCTCGATTCAGACTGTTAGTTAGGGAGTGACTGTTAAAAGTGCATACCGCCAAAAGATAAGG3'
 3'CGCCGCTAUAGCTAAGTCTG5' 3'ACAATCAATCCCTCACTGACAATTTTCACGTATGGCGGTTTTCTATTCC5'
TS35sU
```

non-promoter template

```
 5'GCGGCGATCTCGATTCAGACCGCCCTCTGGTTCTTGGCGGATCCTCCAATGGGGAAGGACACGGACGACC3'
 3'CGCCGCTAUAGCTAAGTCTG5'3'GCGGGAGACCAAGAACCGCCTAGGAGGTTACCCCTTCCTGTGCCTGCTGG5'
TS35sU
```

U = 4-thioUMP

Figure S1. Synthetic promoter templates used in the course of the work.
Transcription start site (+1) is indicated.

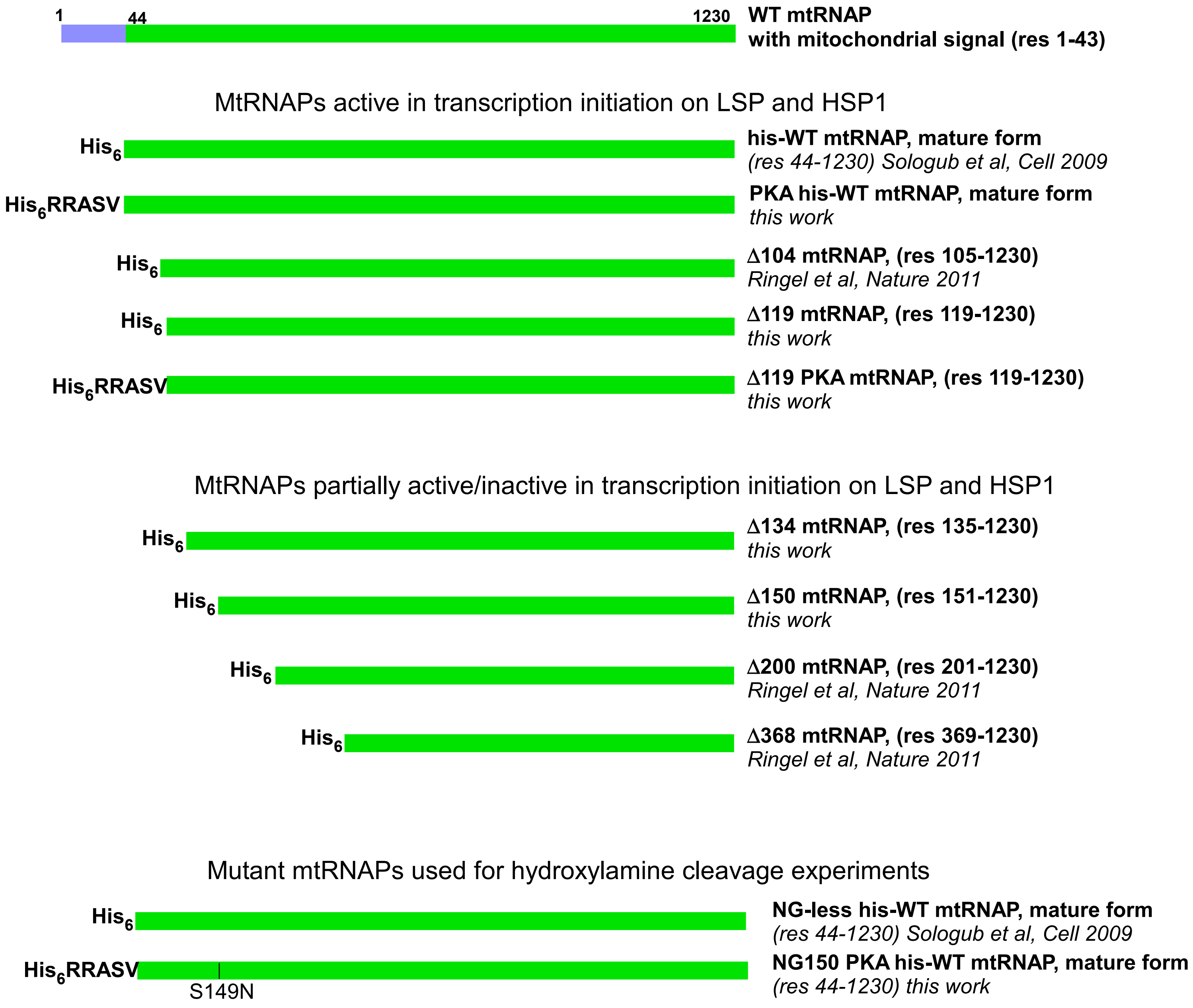

Figure S2. MtRNAP variants used in the course of the work

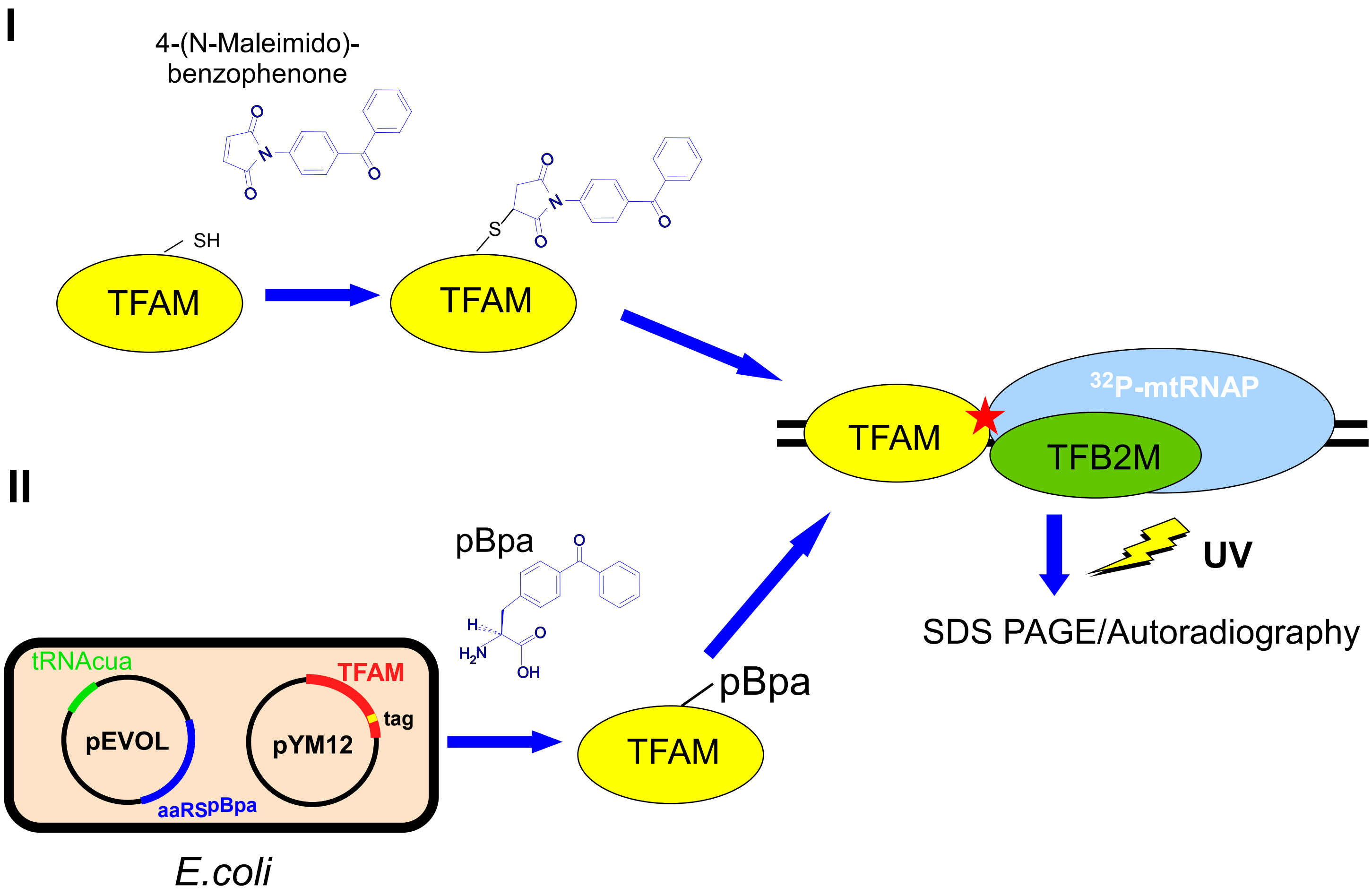

Figure S3. Schematics of the photo cross-linking experiments. Two strategies were used. Purified TFAM containing a single cysteine residue at a defined position was modified using 4-(N-maleimido)-benzophenone, MBP (I). Two naturally occurring cysteine residues (Cys42 and Cys246) that are dispensable for TFAM function[21] were substituted or eliminated to make a cysteine-less TFAM variant that was subsequently used to create a series of single cysteine substitution mutants (Figure S3A and Table S1). Upon modification the initiation complexes were assembled using DNA, ^{32}P-labeled mtRNAP and TFB2M. Alternatively, TFAM was expressed in *E.coli* cells to incorporate a photo reactive amino acid, para benzophenone-phenylalanin, pBpa (II). The pBpa-TFAM was purified and the complexes were assembled as described above.

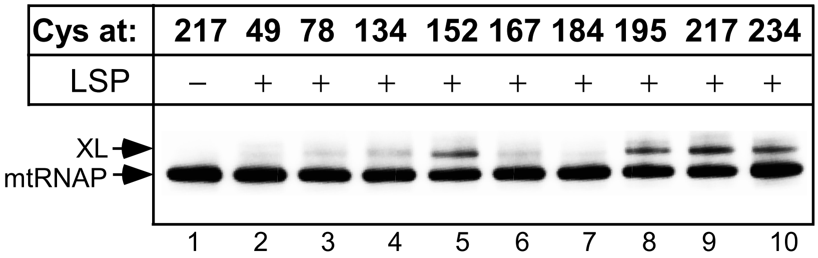

Figure S4. Scanning cross-linking of MBP-modified TFAM and mtRNAP. The pre-initiation complexes were assembled using ^{32}P-labeled mtRNAP (150 nM), 50 nM LSP and 50 nM MBP-modified TFAM having a single cysteine residue at the position indicated, UV irradiated and resolved in SDS-PAGE.

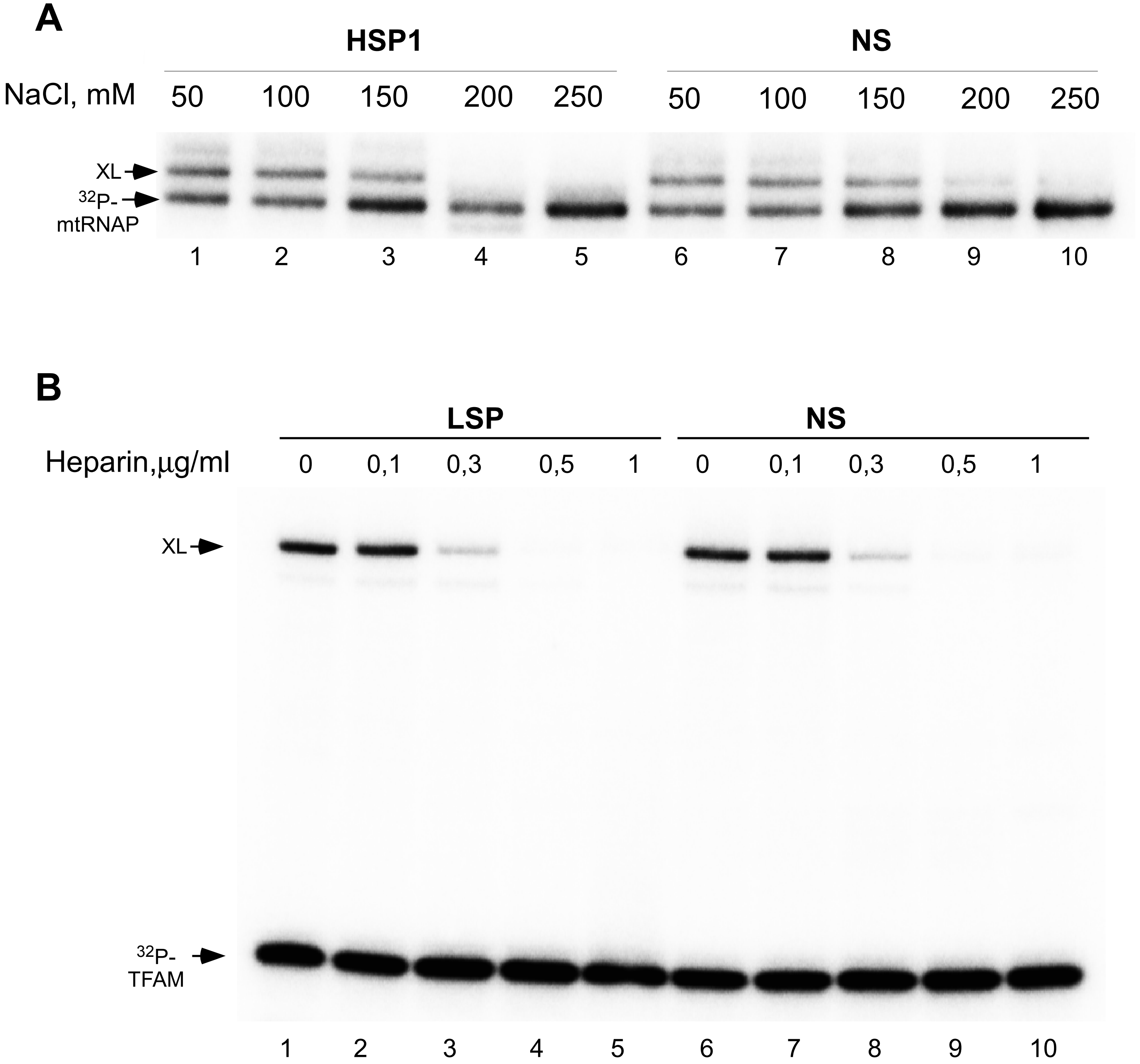

Figure S5. Efficiency of TFAM-mtRNAP cross-linking on LSP, HSP1 and non-specific DNA

A. Efficiency of the cross-linking in salt challenge experiments. The cross-linking was performed using Cys217MBP-TFAM, ^{32}P-labeled Δ119 mtRNAP and HSP1 or non-specific (NS) DNA (80 bps) in the presence of the indicated concentration of NaCl, and analyzed in SDS-PAGE.

B. Efficiency of the cross-linking in heparin challenge experiments. The cross-linking was performed using ^{32}P-labeled Cys217MBP-TFAM, Δ119 mtRNAP and LSP or non-specific DNA (80 bps) in the presence of the indicated concentration of heparin and analyzed in SDS-PAGE.

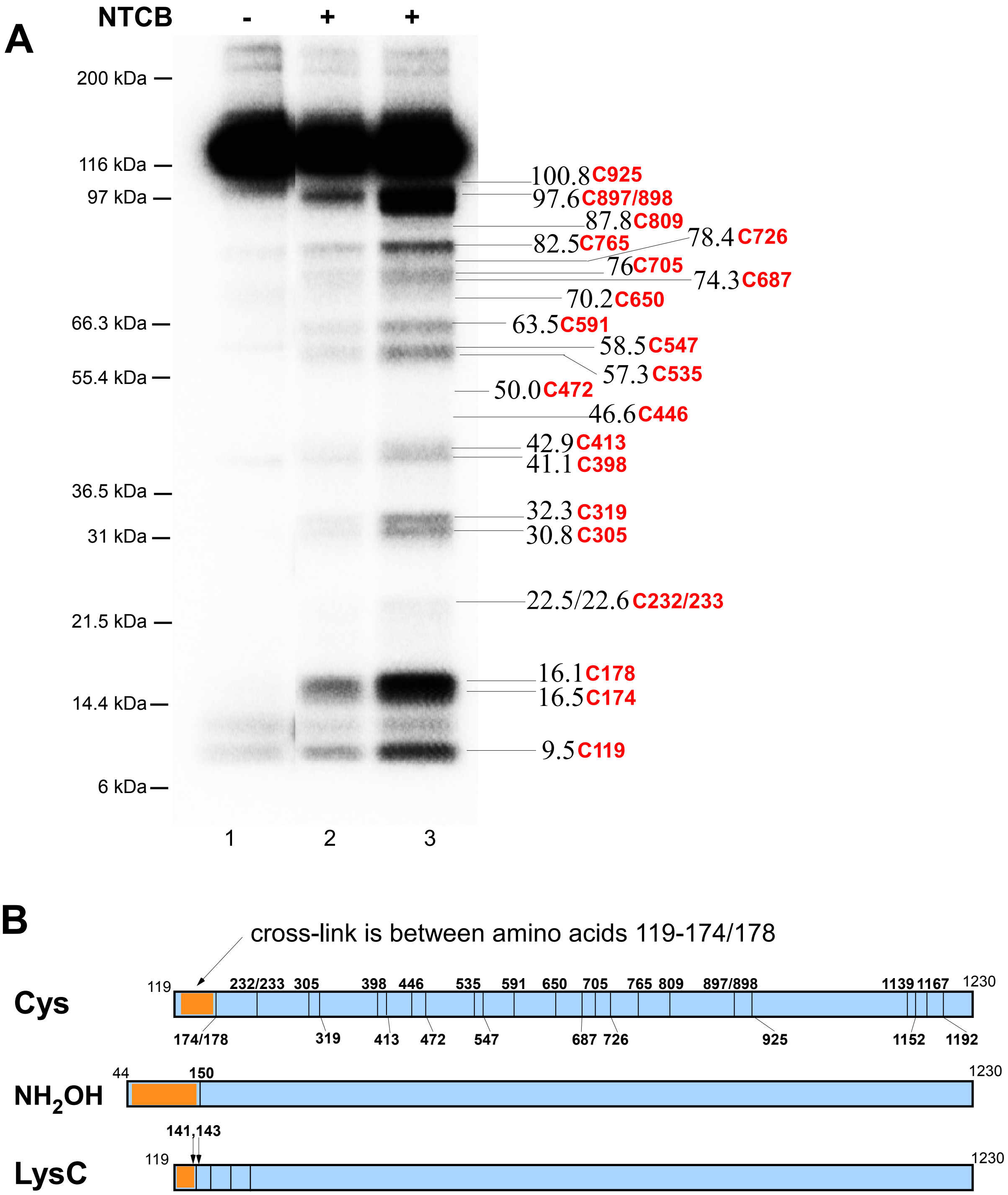

Figure S6. Mapping experiments.

A. Peptide markers generated upon cleavage of ^{32}P-labeled mtRNAP with NTCB. ^{33}P-labeled PKA mtRNAP (44-1230, lane 1) was treated with NTCB for 5 (lane 2) or 10 (lane 3) min. The products of the reaction were resolved in 4-12% MES SDS-PAGE (Invitrogen). Positions of molecular weight markers (Mark 12, Invitrogen) are indicated to left of the gel. Black numbers indicate theoretical molecular weight (in kDa) of the fragments generated by NTCB cleavage, red numbers indicate a cysteine residue at which the cleavage occurred. Cleavage of mtRNAP at cysteine 446 is inefficient and produces a band visible only on an overexposed autoradiogram.

B. Schematics of the mapping experiments using NTCB , hydroxylamine (cleaves mtRNAP variant at position 150) and Lys C (cleaves after lysines).

.

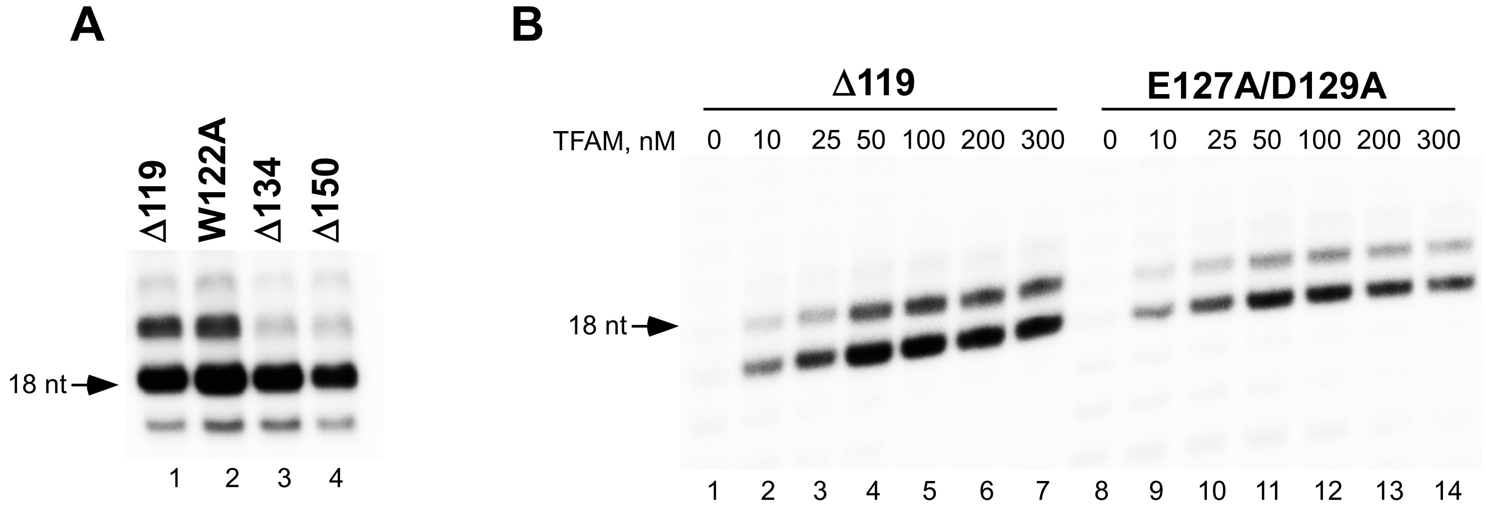

Figure S7. Activity of mutant mtRNAPs in transcription assays.

A. Catalytic activity of mutant mtRNAPs. The mtRNAP mutants were analyzed for transcription activity using bubble LSP template in the absence of TFAM and TFB2M as described previously by Sologub et al[22].

B. E127A/D129A mtRNAP has a reduced transcription activity. Transcription was performed using LSP promoter template (50 nM), TFB2M (50 nM), mtRNAP (50 mM), the indicated amounts of TFAM and NTP mixture (ATP, GTP and UTP) allowing for a synthesis of a 17-18 nt RNA products.

Table S1. Activity of TFAM cysteine mutants used in MBP cross-linking experiments

TFAM[1]	Relative transcription activity[2,3], %
WT (45-246)	100+/-5
del Cys246 (45-245)	103+/-1
C49S	100
Cys-less (C49S, 45-245)	103+/-3
T78C	101+/-3
T122C	ND[4]
S124C	ND
M134C	95+/-4
L152C	102+/-6
A167C	102+/-7
T184C	85+/-6
S195C	100+/-11
S217C	106+/-4
V225C[5]	99+/-6
T234C	97+/-6

[1] All single cysteine substitutions were made in Cys-less TFAM background.

[2] Activity of TFAM mutants was measured using run-off assay. The reactions contained 50 nM LSP template, 50 nM mtRNAP, 50 nm TFB2M and 50-300 nM of TFAM.

[3] All MBP-derivatized TFAM cysteine mutants retained close to the WT TFAM transcription activity (not shown in the table)

[4] ND - activity was not determined

[5] V225C TFAM could not be modified with MBP and was not used in cross-linking experiments.

Table S2. Activity of TFAM variants containing pBpa substitutions

TFAM[1]	Relative transcription activity on LSP[2], %	Relative transcription activity on HSP1[2], %
WT (45-246)	100+/-5	100+/-4
PKA TFAM	100	100
S124pBpa	72+/-3	ND[3]
M127pBpa	46+/-3	ND
S195pBpa	98+/-4	ND
E198pBpa	93+/-3	ND
S217pBpa	95+/-3	91+/-3
E220pBpa	95+/-7	ND
E224pBpa	87+/-6	ND
G226pBpa	92+/-3	ND
R227pBpa	72+/-3	ND
K228pBpa	49+/-2	53+/-2
R233pBpa	90+/-4	ND
T234pBpa	101+/-5	ND

[1] All pBpa substitution mutants were made in PKA TFAM background.

[2] Activity of TFAM mutants was measured using transcription assay. The reactions contained 50 nM LSP or HSP1 template, 50 nM mtRNAP, 50 nm TFB2M, 50 nM of TFAM and nucleotide mixture as described in Experimental Procedures.

[3] ND- activity was not determined.
